# Cytomegalovirus Lymphadenitis After Anti-CD19 Chimeric Antigen Receptor T-cell (CAR-T): An Underappreciated Etiology of Hypermetabolic Lymphadenopathy in the Post-CAR-T Setting

**DOI:** 10.7759/cureus.100035

**Published:** 2025-12-24

**Authors:** Daniel Rosas, Alissa Cox, Diana Martinez, Amneh Fares, Ihsane Ouansafi, Dante Melendez, Jose Sandoval-Sus

**Affiliations:** 1 Hematology-Oncology, Memorial Healthcare System, Pembroke Pines, USA; 2 Infectious Disease, Memorial Healthcare System, Pembroke Pines, USA; 3 Hematology, Memorial Healthcare System, Pembroke Pines, USA; 4 Hematopathology, Memorial Healthcare System, Pembroke Pines, USA; 5 Hematology-Oncology, Moffitt Cancer Center, Memorial Healthcare System, Pembroke Pines, USA

**Keywords:** allogeneic stem cell transplant recipients, cytomegalovirus (cmv), hemato-oncology, non-hodgkin's lymphoma, oncolog

## Abstract

Chimeric antigen receptor T-cell (CAR-T) is a revolutionary type of immunotherapy that genetically engineers a patient's own immune T-cells to recognize and attack cancer cells. This type of therapy has transformed the treatment of relapsed/refractory large B-cell lymphoma (LBCL). However, profound immunosuppression may lead to opportunistic infections that could resemble relapse in rare situations. Cytomegalovirus (CMV) reactivation and clinical infection are well recognized after hematopoietic stem cell transplantation but are less studied in the post-CAR-T setting. We describe a 56-year-old woman with primary refractory stage IV diffuse LBCL, not otherwise specified, treated with axicabtagene ciloleucel. She initially achieved a complete remission (CR), but at six months s/p CAR-T, she developed constitutional symptoms and non-tender palpable lymphadenopathy. Positron emission tomography (PET) and computed tomography (CT) demonstrated hypermetabolic cervical and axillary lymph nodes, as well as focal uptake in the appendix. A biopsy did not show relapsed lymphoma but did reveal classic CMV inclusions, positive CMV immunohistochemistry, and high-level tissue CMV DNA despite negative plasma PCR, with a final diagnosis of CMV lymphadenitis. Symptoms resolved with a three-week course of valganciclovir, and follow-up imaging showed near-complete resolution of the disease. CMV lymphadenitis is a rare but important differential diagnosis of hypermetabolic lymphadenopathy in the post-CAR-T setting, which can mimic lymphoma relapse. Tissue biopsy remains essential for accurate diagnosis and to prevent unnecessary oncologic therapy. Clinicians should maintain vigilance for infectious etiologies in this setting, and future studies may clarify whether targeted CMV monitoring is warranted in high-risk patients.

## Introduction

Chimeric antigen receptor (CAR) T-cell therapy provides durable remissions in relapsed/refractory large B-cell lymphoma (LBCL), but it induces prolonged immunosuppression through lymphodepletion, B-cell aplasia, and secondary hypogammaglobulinemia, among other immunosuppressive mechanisms. This increases susceptibility to a variety of opportunistic infections, which increases morbidity and mortality in our patients, impacting post-treatment surveillance and outcomes [1]. Cytomegalovirus (CMV), with a global seroprevalence of over 80%, commonly reactivates in immunocompromised hosts, especially after allogeneic hematopoietic stem cell transplantation (alloHCT) and solid organ transplantation [2,3]. However, its incidence, presentation, and management in CAR-T recipients are less well defined. Unlike in the alloHCT setting, there are no consensus guidelines for CMV prophylaxis or routine surveillance after CAR-T therapy. CMV lymphadenitis, a rare entity characterized by nodal infection and cytopathic inclusions, may appear indistinguishable from lymphoma on imaging. Only histopathology can confirm the diagnosis. We present a case of biopsy-proven CMV lymphadenitis after anti-CD19 CAR-T, initially suspected to be lymphoma relapse.

## Case presentation

**Figure 1:**
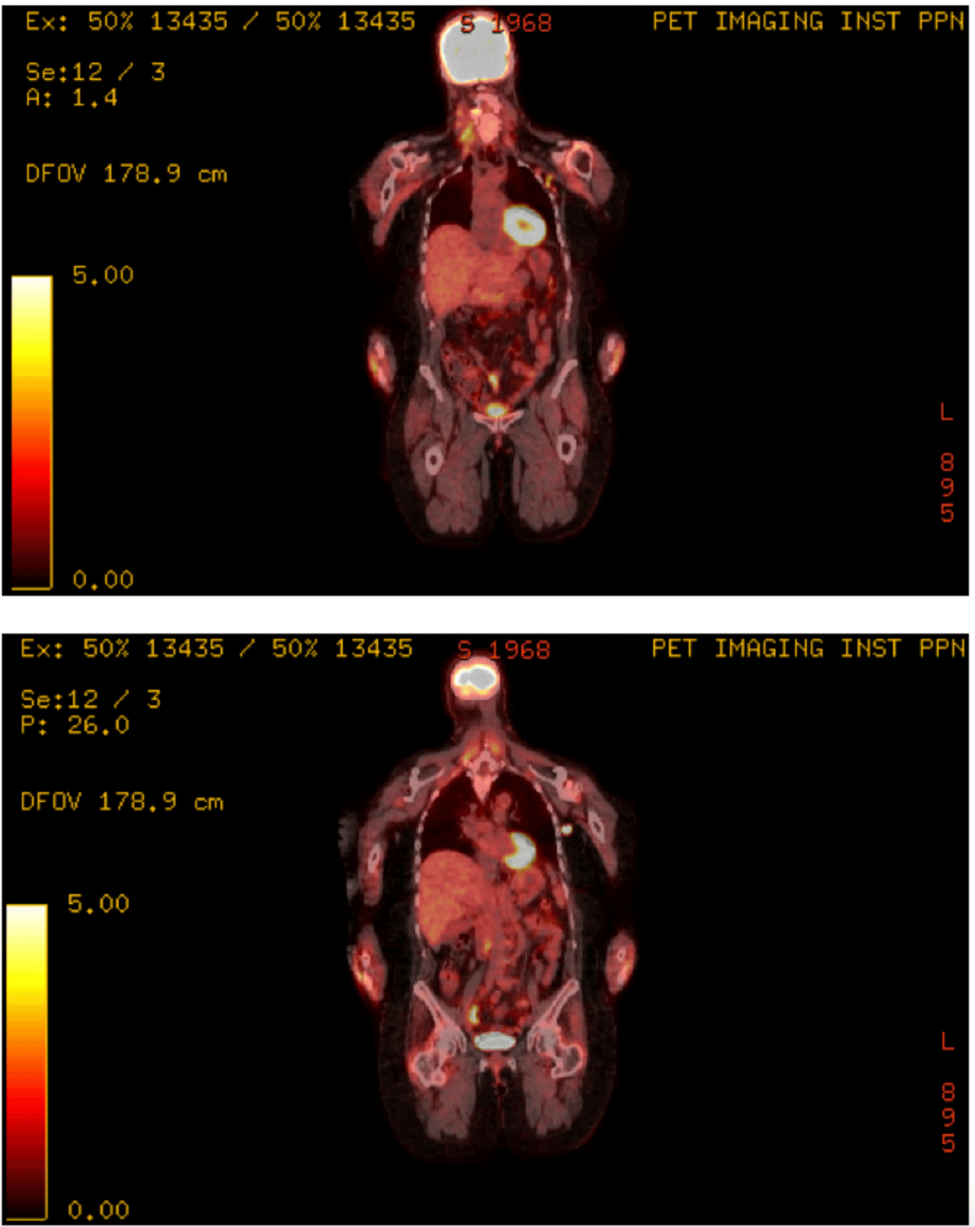
Sequential PET-CT scans. Intense hypermetabolic activity in cervical and left axillary lymph nodes, initially suggestive of relapse.

A 56-year-old woman with stage intravascular diffuse large B-cell lymphoma, not otherwise specified (IV DLBCL NOS) with extranodal involvement, including a bulky left femoral mass that was refractory to six cycles of R-CHOP (rituximab, cyclophosphamide, doxorubicin, vincristine, and prednisone), underwent axicabtagene ciloleucel (Yescarta®) treatment after bridging therapy with radiation therapy to the left femoral mass, followed by lymphodepletion with fludarabine and cyclophosphamide. She achieved a complete remission (CR) based on restaging PET-CT at three months. Six months after CAR-T infusion, she developed generalized malaise, fevers, episodes of nocturnal diaphoresis along with non-tender cervical and axillary lymphadenopathy, and left thigh pain. Restaging PET-CT revealed new enlarged fluorodeoxyglucose (FDG)-avid cervical and axillary lymphadenopathies, as well as focal uptake near the appendix, findings concerning for lymphoma relapse (Figure 1).

**Figure 2:**
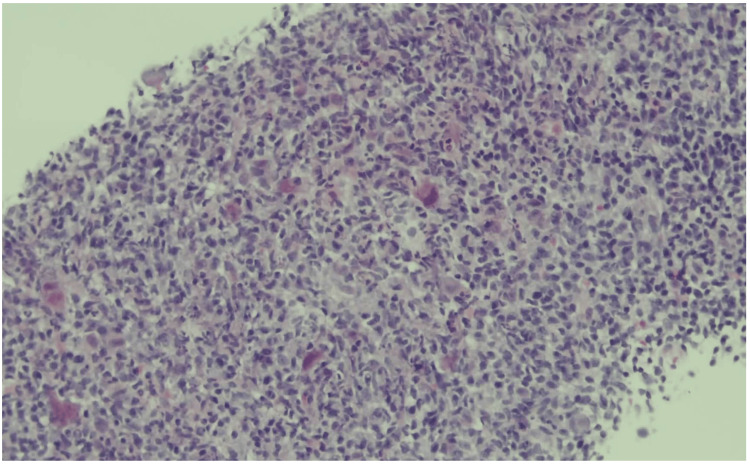
Low-power histopathology of the axillary lymph node (H&E, 20×). Preserved nodal architecture with reactive follicular hyperplasia, prominent germinal centers, and expansion of the paracortex by immunoblasts and monocytoid cells. No necrosis or overt malignant cells are present.

**Figure 3:**
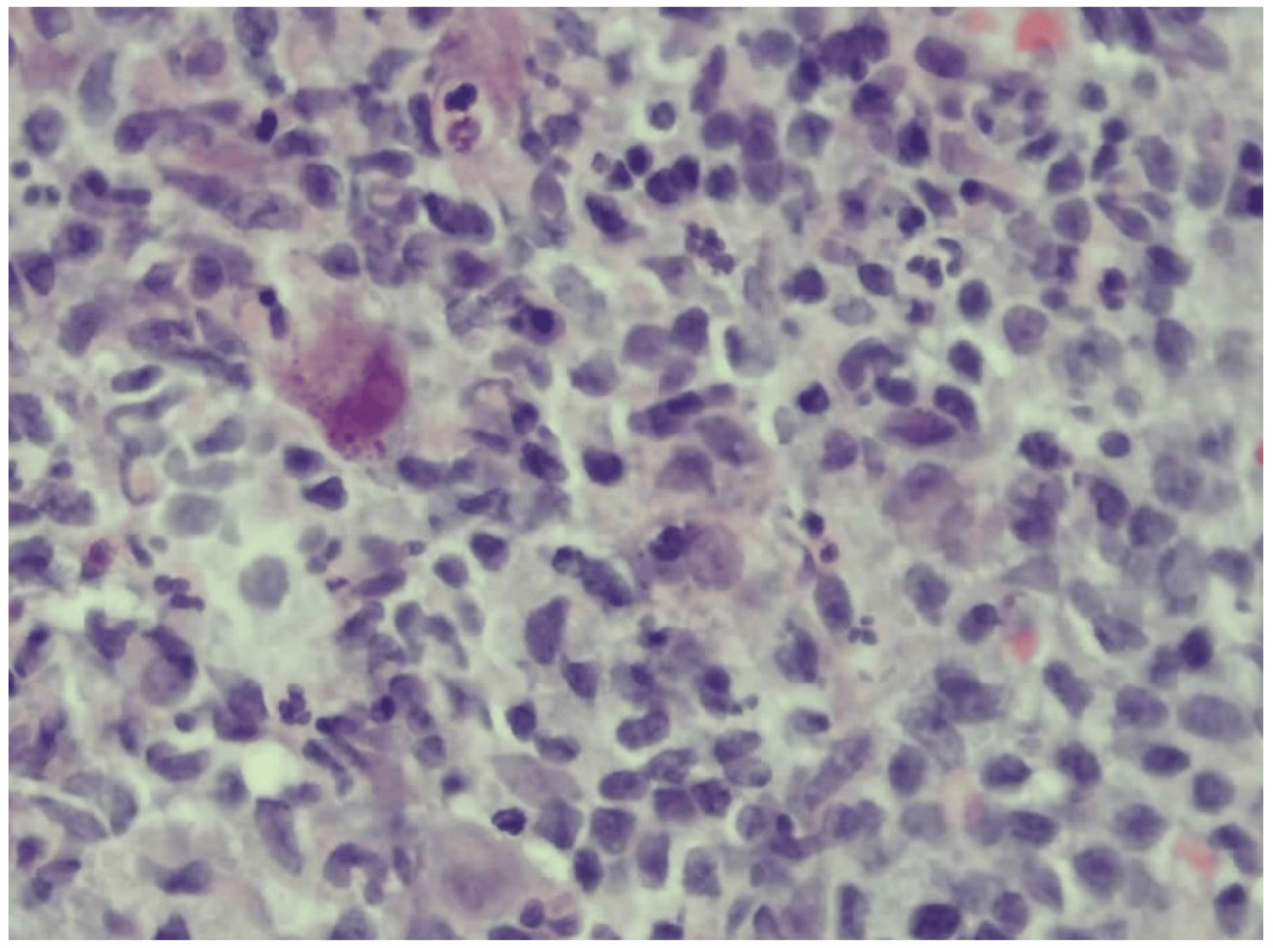
High-power histology (H&E, 40×). Cytomegalic cells with abundant cytoplasm and classic intranuclear “owl’s eye” inclusions are scattered throughout the paracortex and medullary cords, consistent with CMV infection. Background shows a reactive infiltrate of lymphocytes, plasma cells, and histiocytes.

An ultrasound-guided axillary lymph node core-needle biopsy showed preserved architecture with reactive follicular hyperplasia and scattered cytomegalic cells containing owl’s eye intranuclear inclusions (Figures 2-3).

**Figure 4:**
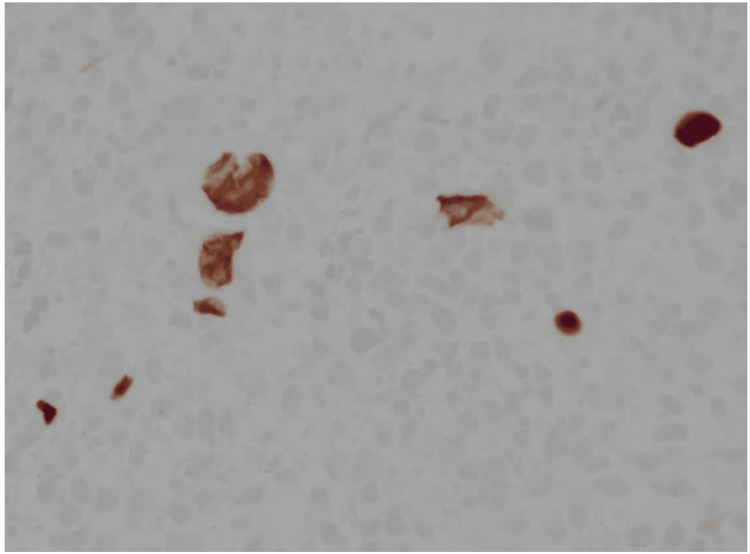
Immunohistochemistry for CMV (40×). Numerous infected cells show strong nuclear positivity, confirming CMV lymphadenitis. Background lymphoid cells remain unstained.

**Figure 5:**
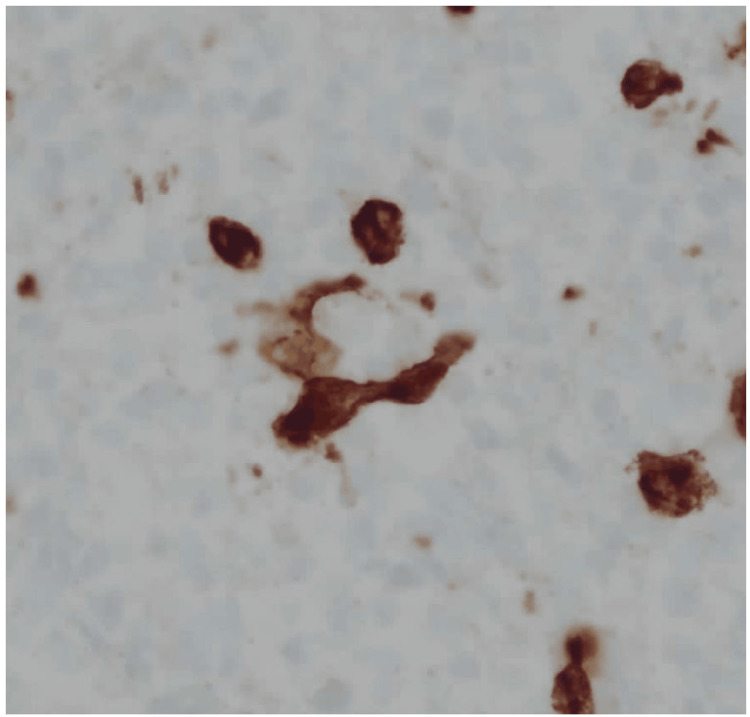
Immunohistochemistry for CD15 (40×). Scattered neutrophils and granulocytes stain positive (internal control), but the CMV-infected inclusion-bearing cells are negative, excluding Hodgkin lymphoma.

Immunohistochemistry was diffusely positive for CMV stain (Figure 4) and negative for lymphoma markers, including CD20, PAX5, CD15, and CD30 (Figure 5).

**Figure 6:**
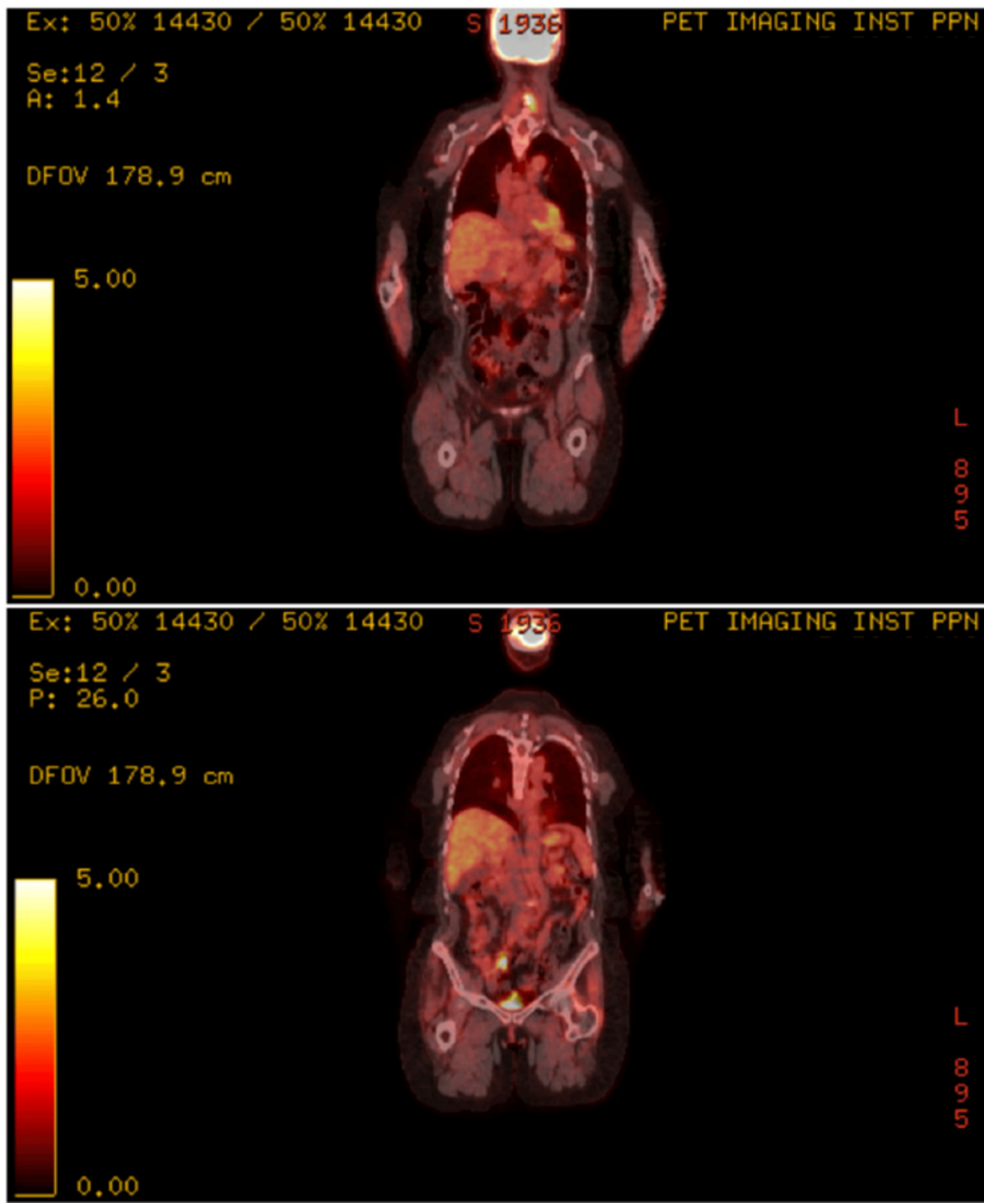
Decreased uptake in the same regions after antiviral therapy, with diffuse tonsillar uptake likely reactive.

**Figure 7:**
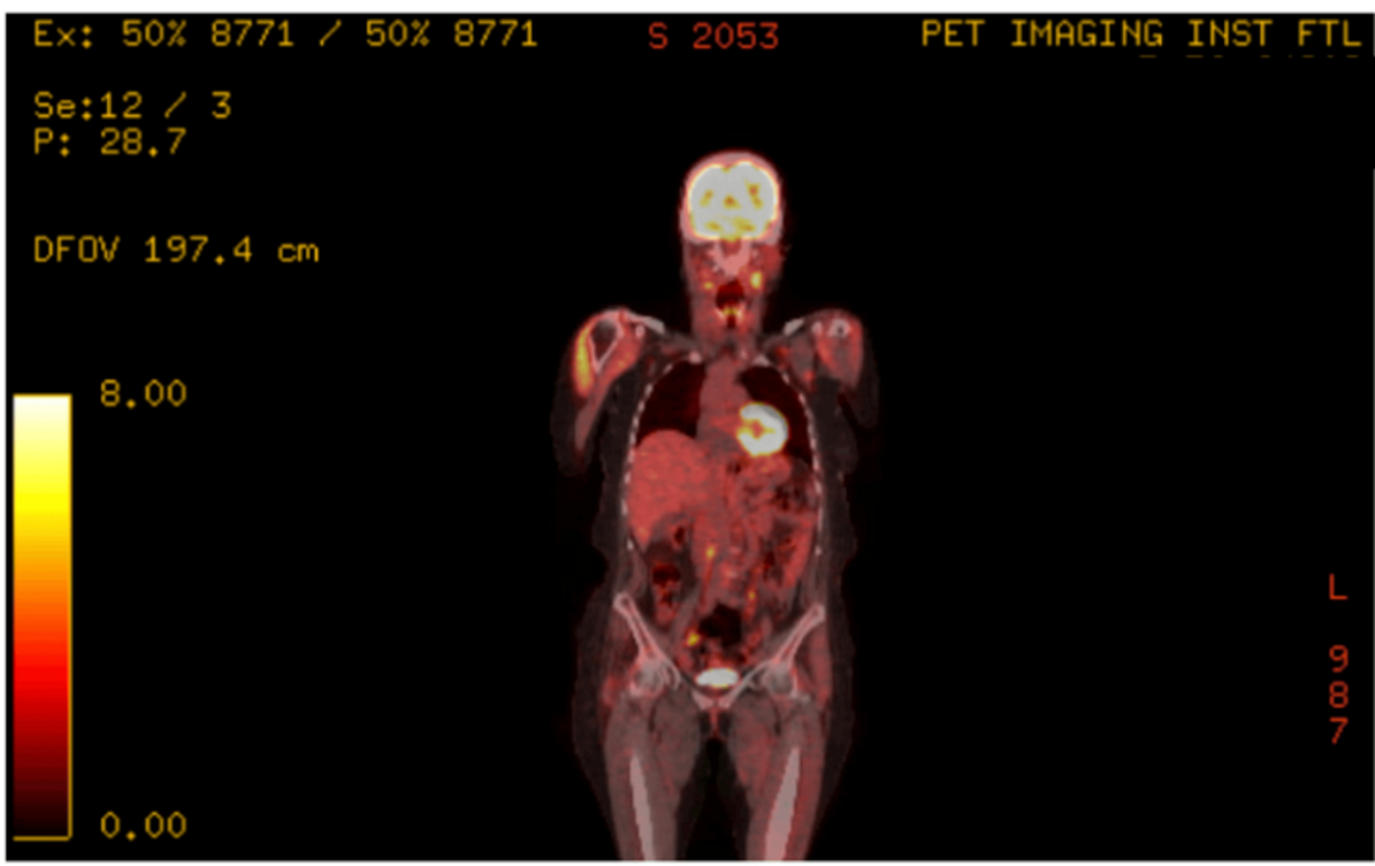
Stable to further improved lymphadenopathy, consistent with resolving CMV lymphadenitis and no new disease.

Tissue PCR confirmed high CMV DNA levels, despite undetectable CMV DNA in plasma via real-time PCR. At the time of this diagnosis, IgG was 1,264 mg/dL, absolute CD4 was 360 cells/uL (CD4%: 17%), and no other co-infections were diagnosed, including Epstein-Barr virus (EBV) infections (EBV-encoded RNA (EBER)-negative on a tissue biopsy). The final diagnosis was CMV lymphadenitis, and she was treated with valganciclovir 900 mg every 12 hours for three weeks, leading to resolution of constitutional and palpable lymphadenopathy. Three months after anti-viral therapy, a PET-CT demonstrated near-complete metabolic resolution of nodes (Figures 6-7), and a restaging PET-CT 12 months post-CAR-T showed CR (DS=2) with resolution of all lymphadenopathies and appendiceal hypermetabolic changes.

## Discussion

This case illustrates that CMV lymphadenitis can closely mimic lymphoma relapse in post-CAR-T patients, based on both clinical and imaging presentations. The combination of constitutional symptoms, palpable and hypermetabolic lymphadenopathy initially suggested progressive disease, but biopsy confirmed the viral infection. Without tissue diagnosis, the patient would face an entirely different prognosis and might have started unnecessary lymphoma-directed therapy.

CAR-T recipients are at risk for opportunistic infections, including viral reactivation due to qualitative and quantitative T and B cell dysfunction, as well as frequent corticosteroid exposure. Although CMV reactivation is common after solid organ and allogeneic HCT, CMV reactivation, clinical infection, and its role in the outcomes of patients treated with CAR-T cell therapy are less studied [3,4]. Our case illustrates that CMV may present as generalized lymphadenitis with negative plasma PCR, emphasizing the need for biopsy even when blood studies are unrevealing. Management of CMV lymphadenitis is not standardized. While observation may suffice in mild cases, symptomatic immunocompromised patients generally require antivirals [5-6]. Our patient responded promptly to valganciclovir, which, as a joint decision with the infectious disease team, underscores the importance of a multidisciplinary care approach for these complex patients to implement tailored therapy according to their needs. As CAR-T use expands for both oncologic and non-oncologic indications, clinicians should remain alert to opportunistic infections that could mimic disease progression or that can dramatically affect the patient’s outcome. We suggest a systematic review of available CMV reactivation and infection post CAR-T cell data, followed by an evidence-based consensus to establish CMV monitoring strategies in high-risk patients, and assess the clinical benefit of early antiviral interventions.

## Conclusions

CMV lymphadenitis is a rare but important mimic of lymphoma relapse in CAR-T cell therapy recipients. PET-CT and CMV plasma PCR may be misleading; therefore, a tissue biopsy remains essential for an accurate diagnosis. Our case demonstrates that timely recognition and antiviral therapy can resolve symptoms and prevent unnecessary oncologic treatment. Clinicians should maintain a broad differential diagnosis for various causes of generalized lymphadenopathy after CAR-T therapy, including both infectious and non-infectious causes, and approach these cases in a multidisciplinary manner to achieve the best outcome.
